# Assessment of early gastric cancer visibility in deep-learning-based virtual indigo carmine chromoendoscopy (with video)

**DOI:** 10.1055/a-2779-0074

**Published:** 2026-01-15

**Authors:** Ayaka Takasu, Sho Suzuki, Yusuke Monno, Masaki Minai, Toshiaki Hirasawa, Hiroyuki Yamamoto, Fumiaki Ishibashi, Toshihiro Nishizawa, Masatoshi Okutomi, Tomohiro Tada

**Affiliations:** 1 Department of Gastroenterology, Cancer Institute Hospital, Japanese Foundation for Cancer Research, Koto-ku, Japan; 2 38259 Department of Gastroenterology, International University of Health and Welfare Ichikawa Hospital, Ichikawa, Japan; 3 13290 Department of Systems and Control Engineering, School of Engineering, Institute of Science Tokyo, Meguro-ku, Japan; 4 625200 Department of Gastroenterology, International University of Health and Welfare Narita Hospital, Narita, Japan; 5 AI Medical Service Inc., Tokyo, Japan; 6 J's Clinic of Gastrointestinal Endoscopy & Proctology, Saitama, Japan

**Keywords:** Endoscopy Upper GI Tract, Diagnosis and imaging (inc chromoendoscopy, NBI, iSCAN, FICE, CLE), Endoscopic ultrasonography, Gastric cancer, Quality and logistical aspects, Image and data processing, documentation

## Abstract

**Background and study aims:**

Indigo carmine chromoendoscopy (IC) enhances diagnosis of early gastric cancer (EGC), but its clinical application is limited by procedure complexity and time. We developed a deep-learning system using a cycle-consistent generative adversarial network (CycleGAN) to generate virtual IC images from white-light endoscopy (WLE) and evaluated visibility of EGC in video-based virtual IC in a pilot study.

**Patients and methods:**

We collected 4,096 endoscopic still images (2,089 WLE, 2,007 real IC) from 262 patients with gastric neoplasms. A CycleGAN model was trained to convert WLE into virtual IC images, and videos with 512 × 512 pixels at 30 frames per second were generated for five EGC cases. For each case, WLE, real IC, and virtual IC videos were prepared and evaluated by 16 endoscopists (6 experts, 10 non-experts). Visibility relative to WLE was rated using a 7-point Likert-type scale (−3 to +3), with positive values indicating improved visibility.

**Results:**

A total of 160 evaluations were performed. Median [IQR] visibility score was 1 [0–2)] for real IC and 0 [−1 to 1] for virtual IC ( *P* < 0.001). In virtual IC, 46.3% of cases achieved a score of +1 or higher. Scores significantly varied by endoscope system ( *P* < 0.001).

**Conclusions:**

Virtual IC improved visibility compared with WLE in nearly half the assessments, although its efficacy did not equal real IC. Optimizing performance for specific endoscope systems may enhance its clinical utility as a practical alternative for improving EGC detection.

## Introduction

Gastric cancer is the fifth most common malignancy and the fifth leading cause of cancer-related mortality worldwide 1 . Prognosis depends largely on early detection, with endoscopic diagnosis playing a central role, as demonstrated by nationwide screening programs in high-incidence countries such as Japan and Korea 2 3 4 . However, early gastric cancer (EGC) frequently presents with subtle or flat morphological changes, making detection challenging. Diagnostic accuracy is influenced by factors such as endoscopist experience, lesion location, and visual conditions 5 6 7 .

Indigo carmine chromoendoscopy (IC) enhances mucosal contrast and has been shown in multiple studies to improve detection of EGC and premalignant lesions compared with standard white-light endoscopy (WLE), with up to a 30% increase in detection 8 9 10 11 . The dye accentuates mucosal surface topography and delineates lesion borders, particularly in flat or depressed-type neoplasms often difficult to identify with WLE alone. Despite its effectiveness, IC has notable limitations, including the need for dye preparation and spraying, variability in staining quality, and the requirement for operator training. Furthermore, the additional examination time may increase patient discomfort and procedural burden.

Recently, deep learning techniques have been increasingly applied to endoscopic imaging. Among these, the cycle-consistent generative adversarial network (CycleGAN) has attracted attention for its ability to perform image-to-image translation using unpaired training data 12 . This method enables the style transfer of standard white-light images into images that mimic the appearance of IC chromoendoscopy, eliminating the need for actual dye application.

We previously developed a CycleGAN-based system capable of generating virtual IC images from WLE still images. This system demonstrated improved visibility for specific lesion types, such as depressed or small lesions 13 . However, in that study, the assessment was limited to still images, which do not reflect the dynamic nature of actual endoscopic practice. In addition, no data were provided comparing virtual and real chromoendoscopy.

Because endoscopic diagnosis occurs in a dynamic environment with continuously changing angles, distances, and lighting conditions, real-time evaluation of visibility is critical for clinical implementation. Therefore, assessing visibility of virtual IC in video format is a necessary step toward practical use.

To address this gap, we developed a novel real-time system using CycleGAN to generate virtual IC images from endoscopic videos. This system enables on-the-fly generation of enhanced images without interrupting the examination workflow. The aim of this study was to evaluate visibility of EGC lesions in virtual IC videos generated using CycleGAN.

## Patients and methods

### Study design

This multicenter, retrospective, observational study was intentionally designed as a pilot feasibility evaluation to assess visibility of EGC in video-based virtual IC generated using CycleGAN. The study employed the paired comparison method, which enables multiple within-lesion and within-observer evaluations, allowing efficient collection of preliminary visibility data from a limited number of consecutive cases.

### Ethics approval and consent

This study protocol was approved by the Institutional Review Board (IRB) of the International University of Health and Welfare on June 25, 2024 (Approval No. 24-Ic-002). The IRB served as the central body overseeing ethical review for all participating sites. All patients provided written informed consent prior to inclusion in the study.

### Generation of virtual IC images and creation of video clips using CycleGAN

This video demonstrates how the same lesion appears when examined using white-light endoscopy, real indigo carmine chromoendoscopy (IC), and virtual IC.Video 1

**Fig. 1:**
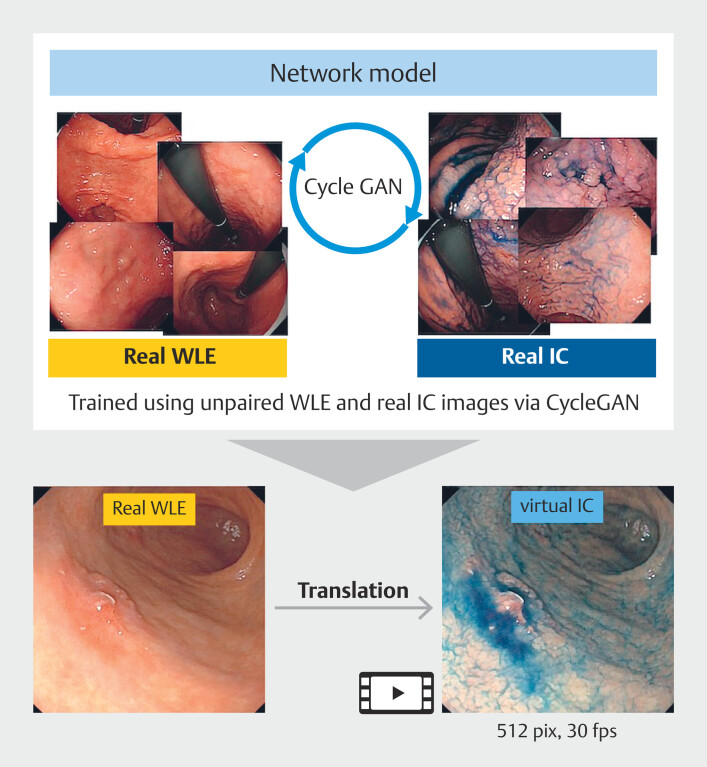
Schema of virtual indigo carmine chromoendoscopy development using CycleGAN. The CycleGAN model was trained using 2,089 white-light endoscopy (WLE) images and 2,007 real indigo carmine chromoendoscopy (IC) images of gastric neoplasms. After training, virtual IC images were generated from WLE images using the trained model. These images were subsequently compiled into video clips for this study.

A schematic overview of the virtual IC image generation process using CycleGAN is presented in Fig. 1 . In our prior study, we demonstrated successful generation of virtual IC images of normal gastric mucosa by applying CycleGAN to unpaired WLE and real IC images. We employed the same methodology to generate virtual IC images for this study using a dataset of WLE and real IC images from patients with gastric neoplasms.

The dataset comprised 4,096 endoscopic still images (2,089 WLE images and 2,007 real IC images) collected from 262 patients with gastric neoplasms, who underwent endoscopic examinations between 2015 and 2024 at three hospitals: Yuri Kumiai General Hospital, International University of Health and Welfare Narita Hospital, and Ichikawa Hospital. Standard- and high-definition endoscopes (GIF-Q260, GIF-Q260J, GIF-XP260N, GIF-H260Z, GIF-XP290N, GIF-H290; Olympus Co., Tokyo, Japan and EG-860R, EG-840T, EG-840N, EG-760R, EG-760Z, EG-740N, EG-L580RD7; Fujifilm Co., Tokyo, Japan) and corresponding endoscopy systems (CV-260 or CV-290; Olympus Co., and VP7000; Fujifilm Co.) were used for all WLE and IC examinations. For IC, 0.1% indigo carmine dye (Alfresa Pharma Corporation, Osaka, Japan) was sprayed directly onto the lesion area.

To address variations in image resolution caused by differences in endoscope models, preprocessing and model training were performed separately for each endoscopic system. All images were resized prior to training: for the CV-290 system, 911 WLE and 722 IC images (total = 1,633) were resized to 512 × 448 pixels; for the VP-7000 system, 1,178 WLE and 1,285 IC images (total = 2,463) were resized to 600 × 600 pixels. Separate CycleGAN models were trained for each system using identical network architecture and hyperparameters to account for manufacturer-specific imaging characteristics. The CycleGAN architecture followed the original design, consisting of nine ResNet blocks for the generator and three convolutional layers of a PatchGAN discriminator for the discriminator network. The cycle consistency loss weight (λ _cyc_ ) and identity loss weight (λ _idt_ ) were set to 10 and 5, respectively, in line with our previous work.

To optimize for manufacturer-specific imaging characteristics, CycleGAN models were trained separately for image datasets derived from CV260/290 and VP7000 endoscopes. Each model was trained independently using the same network architecture and hyperparameters.

The network was trained for 100 to 200 epochs on a single NVIDIA GeForce RTX 3090 GPU, requiring approximately 48 hours.

The trained model was then used to generate virtual IC images from WLE still images. Subsequently, video clips of virtual IC were created for evaluation, standardized to a resolution of 512 × 512 pixels and 30 frames per second (fps) ( Video 1 ).

### Evaluation setting and outcome

A total of 15 uncompressed, original-quality endoscopic video clips were prepared, comprising one high-definition WLE clip, one real IC clip, and one virtual IC clip for each of five cases. Five consecutive EGC lesions treated endoscopically at the International University of Health and Welfare Ichikawa and Narita Hospital between July and August 2024 were selected based on availability of high-quality WLE and real IC videos captured from comparable viewpoints. Virtual IC videos were then generated from these WLE clips using the trained CycleGAN model.

**Fig. 2:**
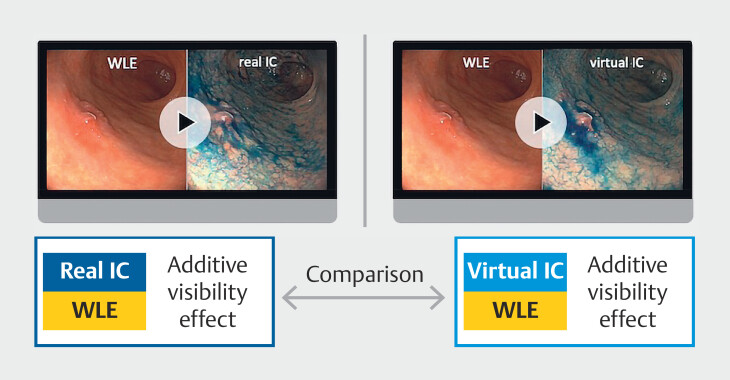
Concept of visibility assessment comparing real and virtual indigo carmine chromoendoscopy relative to white-light endoscopy. Each evaluator rated the additive visibility effect of real and virtual indigo carmine chromoendoscopy (IC) by comparing each to the corresponding white-light endoscopy (WLE) video, using a 7-point scale from −3 (much worse) to +3 (much better), with 0 indicating no difference.

Three cases were examined using the VP7000 system and two cases were examined using the CV-290 system. Each video clip was 20 seconds in length. The clips were evaluated by 16 endoscopists—six experts certified by the Japan Gastroenterological Endoscopy Society and 10 non-experts—who completed all assessments. For each of the five cases, evaluators first viewed the WLE video, followed by the corresponding real IC video, and assessed the additive visibility effect of real IC relative to WLE. They then viewed the same WLE video, followed by the corresponding virtual IC video, and assessed the additive visibility effect of virtual IC relative to WLE ( Fig. 2 ).

Visibility was assessed using the Pair Comparison method, in accordance with recommendations from the International Telecommunication Union Radiocommunications Sector/Telecommunication Standardization Sector (ITU-T/R) for subjective video quality assessment 14 15 . The ITU, a specialized agency of the United Nations, is responsible for establishing international standards for telecommunications and has issued recommendations for video quality assessment under ITU-T/R.

Evaluations were conducted on 32-inch 4K resolution monitors in a darkened room, with up to three physicians present per session. Evaluators were positioned 1.0 to 1.3 meters from the monitor, within 30 degrees of the center of the screen. The primary outcome was the additive effect of real or virtual IC videos compared with WLE videos on visibility of EGC. Visibility was assessed using a 7-point scale, with WLE as the reference (score 0) ( Table 1 ). Positive scores indicated better visibility in the IC video compared with WLE: +1 (slightly better), +2 (better), and +3 (much better). A score of 0 indicated no difference in visibility. Negative scores indicated worse visibility in the IC video compared to WLE: −1 (slightly worse), −2 (worse), and −3 (much worse).

**Table:** **Table 1** Visibility score scale for evaluating real or virtual indigo carmine chromoendoscopy compared to white-light endoscopy.

Score	Definition
+ 3	Much better than WLE
+ 2	Better than WLE
+ 1	Slightly better than WLE
+ 0	About the same
− 1	Slightly worse than WLE
− 2	Worse than WLE
− 3	Much worse than WLE
Visibility of real or virtual indigo carmine chromoendoscopy was evaluated relative to white-light endoscopy using a 7-point scale ranging from −3 (much worse) to +3 (much better), with 0 indicating no difference. WLE, white-light endoscopy.	

Subgroup analyses were performed based on the endoscopy system (VP7000 vs CV-290) and evaluator expertise (experts vs non-experts).

### Statistical analysis

Visibility scores were treated as continuous variables and expressed as medians with interquartile range (IQR). Statistical significance was defined as *P* < 0.05. All statistical analyses were performed using SPSS Statistics version 29.0.2.0 (IBM Corp., Armonk, New York, United States).

## Results

Five EGC lesions were analyzed (Supplementary Table 1). All patients were male, with a median age of 70 years (range, 47–75 years). Macroscopic types included 0-IIc (n = 3), 0-IIa (n = 1), and 0-IIb (n = 1). Tumor size had a median diameter of 11 mm (range, 3–25 mm). Histologically, four lesions were differentiated-type and one was an undifferentiated-type carcinoma; all lesions were non-ulcerated.

**Fig. 3:**
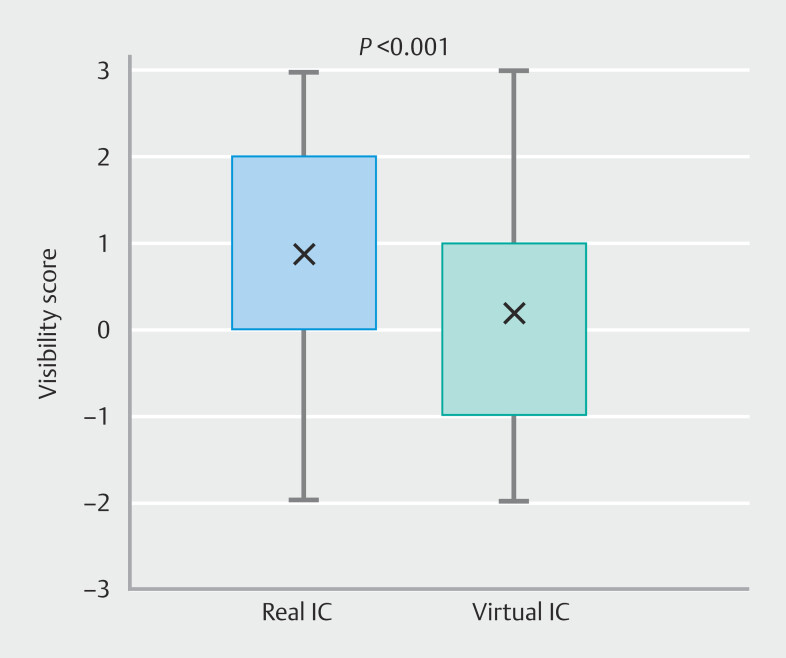
Comparison of visibility scores for early gastric cancer between real and virtual indigo carmine chromoendoscopy. Box plots show the distribution of visibility scores assessed by endoscopists. The median [IQR] score was significantly higher for real IC than for virtual IC ( *P* < 0.001). The visibility score indicates the additive value of visibility compared to white-light endoscopy (WLE).

In total, 160 evaluations were completed, comprising 80 assessments for real IC and 80 for virtual IC. The median [IQR] visibility scores were 1 [0 to 2] for real IC and 0 [−1 to 1] for virtual IC, showing a significant difference ( *P* < 0.001) ( Fig. 3 ). The difference between real IC and virtual IC was statistically significant (Z = −3.207, *P* < 0.001), corresponding to a paired-samples effect size (r = 0.359, moderate).

Among the virtual IC cases, 37 (46.3%) (95% confidence interval [CI] 35.8–57.1) had a visibility score ≥ +1, whereas 52 (65.0%) (95% CI 54.1–74.6) of real IC evaluations were rated ≥ +1. When the threshold was raised to ≥ +2, responder rates were 17.5% (95% CI 10.7–27.3) for virtual IC and 30.0% (95% CI 21.1–40.8) for real IC. To illustrate lesion-level variability, Supplementary Fig. 1 presents per-lesion median visibility scores for both modalities. This complements Supplementary Table 2, which summarizes detailed per-lesion and per-rater score distributions and highlights interrater variability as well as lesion-specific heterogeneity.

### Subgroup analysis by endoscopy system and endoscopic expertise

**Fig. 4:**
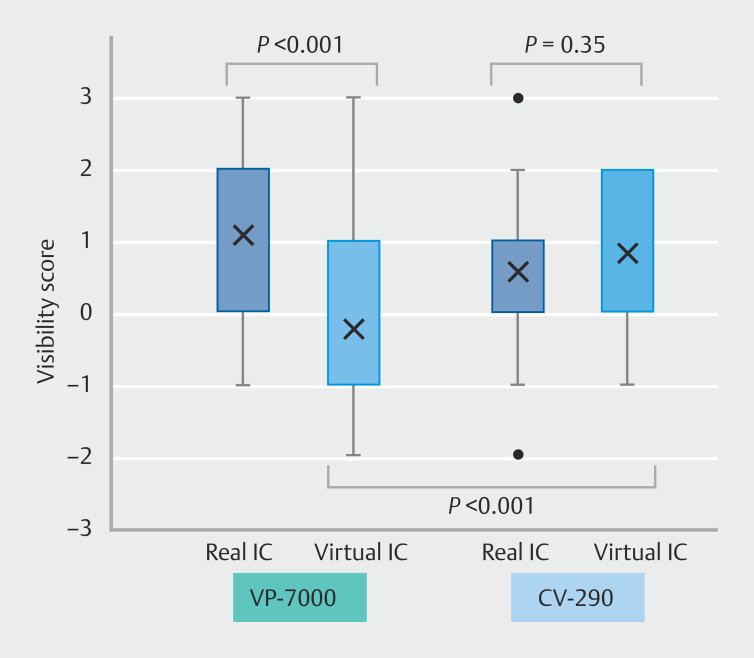
Comparison of visibility scores by endoscopy system. Subgroup analysis according to endoscopy system (VP-7000 vs CV-290). Real IC showed significantly higher scores than virtual IC in the VP-7000 group (P < 0.001), whereas no significant difference was observed in the CV-290 group (P = 0.35). The virtual IC score was significantly higher with the CV-290 than with the VP-7000 (P < 0.001). The visibility score represents the additive value compared with WLE.

Among the 160 evaluations, 64 were for the CV-290 system and 96 for the VP7000 system. In virtual IC videos, visibility scores were significantly higher with the CV-290 system compared with the VP7000 (median [IQR] 1 [0–2] vs. −1 [−1 to 1], *P* < 0.001). Within each system, the CV-290 showed no significant difference between real IC and virtual IC ( *P* = 0.35), whereas the VP7000 showed significantly lower scores for virtual IC compared with real IC ( *P* < 0.001) ( Fig. 4 ). Visibility scores for virtual IC videos did not differ significantly between evaluations performed by experts (30 evaluations) and those by non-experts (50 evaluations) (median [IQR] for both: 0 [−1 to 1], *P* = 0.89).

## Discussion

This pilot study evaluated visibility of EGC using virtual IC images generated from WLE with CycleGAN, presented in video format. Although the improvement in visibility with virtual IC was limited and significantly lower than that for real IC, 46.3% of lesions still achieved a visibility score of +1 or higher. This suggests that nearly half the lesions demonstrated improved visibility with virtual IC compared with standard WLE, indicating its potential as a supportive tool for diagnosing EGC.

To the best of our knowledge, this is the first study to evaluate visibility of EGC using CycleGAN-generated virtual IC in a video-based format. Previous studies have evaluated lesion visibility using virtual chromoendoscopy with still images. For example, Sato et al. applied virtual IC to peroral cholangioscopy and compared delineation of surface structures, microvessels, and lesion margins across white-light, narrow-band, and virtual IC images 16 . Similarly, Toya et al. from our group developed virtual Lugol chromoendoscopy for detection of superficial esophageal squamous cell carcinoma and conducted evaluations based on still images 17 . In contrast, the present study used a video-based format, enabling a more realistic assessment of lesion visibility and providing foundational evidence for the potential clinical application of virtual IC in the evaluation of EGC.

The difference in visibility scores between real and virtual IC appeared to vary depending on the endoscopic system used. In subgroup analyses, virtual IC with the CV-290 system achieved a similar level of lesion visibility to real IC, whereas visibility scores for virtual IC were significantly lower with the VP7000 system. This discrepancy may be attributed to differences in image acquisition methods and internal imaging processes in each endoscope system. For example, each system employs a different image sensor and may apply its own image processing pipeline, such as noise reduction and contrast enhancement, which are not disclosed to end users. These factors contribute to variations in image characteristics, such as resolution, brightness, color contrast, and noise levels, which could impact overall performance of image translation by CycleGAN. Further analysis of the performance gap between different endoscope systems will be required in future work.

IC dye offers several advantages, such as clearer lesion demarcation and enhanced visualization of mucosal structures, thereby improving diagnostic accuracy compared with WLE. However, its use requires dye preparation, spraying, and subsequent washing, which can be time-consuming and labor-intensive. In regions with a high incidence of gastric cancer, particularly in countries such as Japan and Korea, where endoscopic screening is widely implemented, these additional steps can present significant operational challenges, especially in high-throughput screening settings where numerous cases must be managed within limited time frames. Consequently, routine use of real IC may be restricted, potentially limiting diagnostic benefits that could otherwise be achieved. If virtual IC can be implemented in clinical practice, it could enable endoscopists to repeatedly access the benefits of dye-based imaging during procedures without additional effort, offering a promising tool to enhance lesion visibility without compromising procedure efficiency. Furthermore, it may contribute to standardization of diagnostic quality across institutions.

Despite the potential advantages of assessing virtual IC performance using video images, this study has certain limitations, reflecting its focus on rapid development of the system at this early stage. First, it evaluated a small number of recorded videos and was unable to assess performance of virtual dye images in actual patients. Second, visibility was quantified using a 7-point subjective scoring system, which may be influenced by individual evaluator experience and perception, potentially limiting objectivity and reproducibility.Third, all evaluators viewed WLE videos before real IC or virtual IC videos in a fixed sequence. Although this design ensured a consistent reference-based comparison, it may have introduced order or memory bias. Full randomization was not applied because this was a pilot feasibility study aimed at obtaining efficient preliminary evaluations. Finally, this study did not assess detection rates or boundary delineation accuracy. Objective evaluation of these parameters requires a large number of cases and expert annotations, which was not feasible at this early stage of system development.

Nevertheless, the present findings may serve as a foundation for further development and clinical validation of virtual IC. With continued improvements in imaging conditions and system-specific optimization, virtual IC has the potential to enhance diagnostic performance in real-world clinical practice. Building on these preliminary findings, larger-scale prospective human studies will be necessary to validate reproducibility and generalizability of the results. Such studies will be crucial for confirming the diagnostic impact of virtual IC and for establishing system-specific optimization strategies in real clinical settings.

## Conclusions

In conclusion, a virtual IC based on a deep learning model provided an additive visibility effect for EGC over WLE in nearly half the assessments. However, its performance did not reach the level of real IC and depended on the endoscopic system used. By improving the performance of virtual IC, it may serve as a useful alternative to real IC, supporting its potential clinical utility in enhancing visibility of EGC.

## Supplementary Material

Supplementary Material Zusatzmaterial
