# Supplementary material for: Assessment of early gastric cancer visibility in deep-learning-based virtual indigo carmine chromoendoscopy (with video)
Source: Endosc Int Open. 2026 Jan 15;14:a27790074. doi: 10.1055/a-2779-0074 (PMC12817183; doi:10.1055/a-2779-0074)

Supplementary Table 1 Clinicopathological characteristics of the five gastric cancer cases evaluated.

Case	Age	Sex	Atrophy*	System	Location†	Tumor circumference‡	Macroscopic type	Depth	Size (mm)	Histology	UL§
1	70	M	C-3	VP-7000	M	Posterior wall	0-IIc	T1a	18×15	por2 >sig	Absent
2	75	M	O-1	VP-7000	L	Anterior wall	0-IIc	T1b2	25×18	tub2	Absent
3	75	M	O-1	VP-7000	M	Lesser curvature	0-IIb	T1a	3×3	tub1	Absent
4	47	M	C-2	CV-290	U	Greater curvature	0-IIc	T1a	11×5	tub2	Absent
5	61	M	C-2	CV-290	M	Lesser curvature	0-IIa	T1a	6×6	tub1	Absent

*Atrophy grade was classified according to the Kimura–Takemoto classification (C-2, C-3 = closed type; O-1 = open type).
†Location indicates the longitudinal site of the stomach: U, upper third; M, middle third; L, lower third.
‡Tumor circumference denotes the circumferential site of the stomach: lesser curvature, greater curvature, anterior wall, or posterior wall.
§UL indicates presence of ulcerative findings or ulcer scars.

Supplementary Table 2 Number of evaluators assigning each visibility score for each lesion.

Case	System	Modality	Visibility score -3	-2	-1	0	+1	+2	+3	Median [IQR]
1	VP-7000	real IC	0	0	1	3	6	5	1	+1 [+1 to +2]
		virtual IC	0	3	7	0	4	0	2	-1 [-1 to +1]
2	VP-7000	real IC	0	0	2	2	3	6	3	+2 [+1 to +2]
		virtual IC	0	1	9	0	3	1	2	-1 [-1 – +1]
3	VP-7000	real IC	0	0	1	5	8	1	1	+1 [0 to +1]
		virtual IC	0	2	6	6	2	0	0	0 [-1 to 0]
4	CV-290	real IC	0	0	0	3	6	6	1	+1 [+1 to +2]
		virtual IC	0	0	4	1	4	7	0	+1 [0 to +2]
5	CV-290	real IC	0	2	4	5	5	0	0	0 [-1 to +1]
		virtual IC	0	0	2	2	10	2	0	+1 [0 to +1]

Number of evaluators assigning each visibility score (−3 to +3) for each lesion under real and virtual indigo carmine chromoendoscopy (IC).
Median [IQR] values summarize visibility scores across 16 endoscopists.
IQR, interquartile range.

Supplementary Fig. 1 Per-lesion median visibility scores for real and virtual indigo carmine chromoendoscopy. Each pair of boxes represents one lesion (Cases 1–5) evaluated by 16 endoscopists, with white-light imaging (WLI) as the reference (0). Boxes show interquartile ranges with median lines; whiskers indicate minimum and maximum values. This figure visualizes lesion-level variability and complements **Supplementary Table 2**.

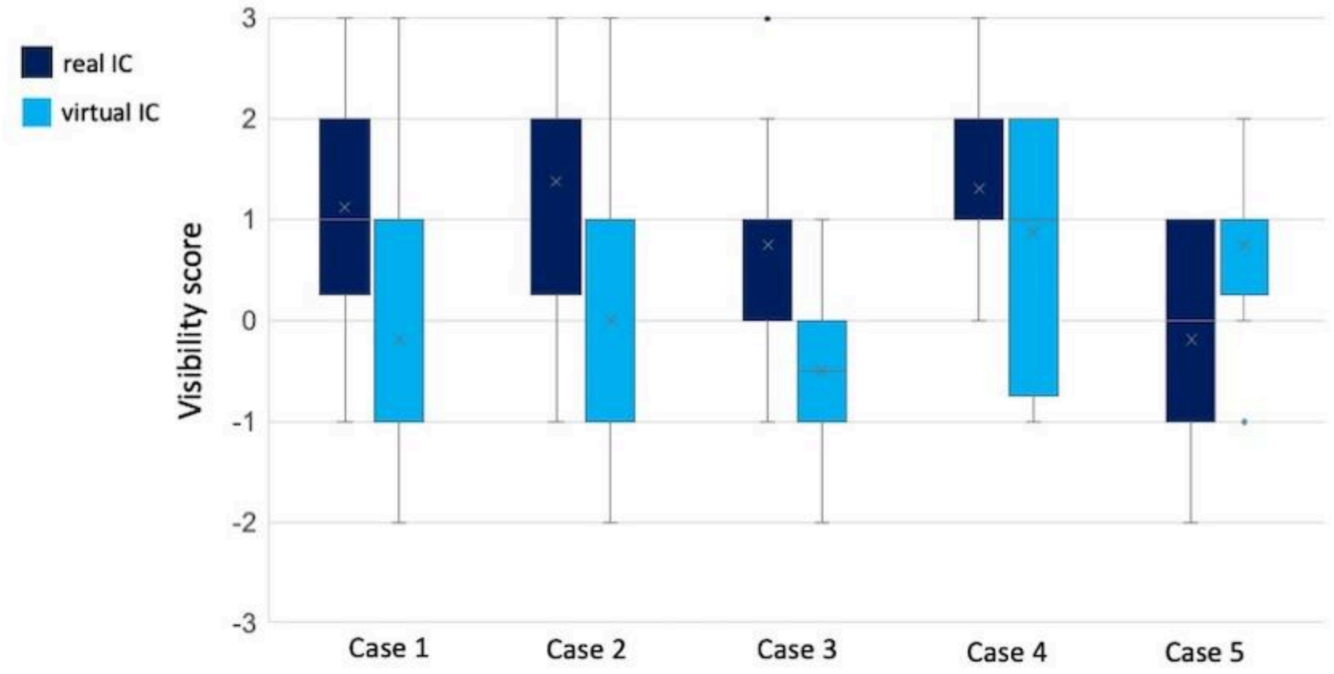

Supplement: Supplementary file 2 — Supplementary Material [file 10-1055-a-2779-0074_27803222.pdf]
